# Bioprintability: Physiomechanical and Biological Requirements of Materials for 3D Bioprinting Processes

**DOI:** 10.3390/polym12102262

**Published:** 2020-10-01

**Authors:** Andrea S. Theus, Liqun Ning, Boeun Hwang, Carmen Gil, Shuai Chen, Allison Wombwell, Riya Mehta, Vahid Serpooshan

**Affiliations:** 1 Department of Biomedical Engineering, Emory University School of Medicine and Georgia Institute of Technology, Atlanta, GA 30322, USA; andrea.theus@emory.edu (A.S.T.); liqun.ning@emory.edu (L.N.); boeun.hwang@emory.edu (B.H.); carmen.julia.gil@emory.edu (C.G.); schen644@gatech.edu (S.C.); awombwel3@gatech.edu (A.W.); 2 Department of Biology, Emory University, Atlanta, GA 30322, USA; riya.mehta@emory.edu; 3 Department of Pediatrics, Emory University School of Medicine, Atlanta, GA 30322, USA; 4 Children’s Healthcare of Atlanta, Atlanta, GA 30322, USA

**Keywords:** bioprinting, printing fidelity, bioink, hydrogel, extrusion, printability, bioprintability, tissue engineering

## Abstract

Three-dimensional (3D) bioprinting is an additive manufacturing process that utilizes various biomaterials that either contain or interact with living cells and biological systems with the goal of fabricating functional tissue or organ mimics, which will be referred to as bioinks. These bioinks are typically hydrogel-based hybrid systems with many specific features and requirements. The characterizing and fine tuning of bioink properties before, during, and after printing are therefore essential in developing reproducible and stable bioprinted constructs. To date, myriad computational methods, mechanical testing, and rheological evaluations have been used to predict, measure, and optimize bioinks properties and their printability, but none are properly standardized. There is a lack of robust universal guidelines in the field for the evaluation and quantification of bioprintability. In this review, we introduced the concept of bioprintability and discussed the significant roles of various physiomechanical and biological processes in bioprinting fidelity. Furthermore, different quantitative and qualitative methodologies used to assess bioprintability will be reviewed, with a focus on the processes related to pre, during, and post printing. Establishing fully characterized, functional bioink solutions would be a big step towards the effective clinical applications of bioprinted products.

## 1. Introduction

Three-dimensional (3D) bioprinting is an additive manufacturing technique, used to create complex tissue and organ structures that can support live cells and other biological factors. Several bioprinting strategies have been explored for the main goal of achieving viable functional constructs for tissue engineering applications [1]. Of these strategies, extrusion-based bioprinting has been the most applicable in the field of tissue engineering due to its facility in printing bioactive bioinks [2]. Other modalities such as inkjet, laser, and stereolithography-based bioprinting have been developed and increasingly used in tissue engineering applications over the past decade, but they typically introduce some complex functionality and at times run at a higher cost than the extrusion technique [3]. 

Inkjet bioprinting is a non-contact printing modality that deposits small ink droplets into a predetermined location driven by thermal or piezoelectric actuation [4]. This method is relatively fast and offers the high-resolution printing of single cells. Laser-based printing has the ability to print biological material such as cells, DNA, and peptides at a relatively high resolution at the microscale [5,6]. However, due to the pulsed laser source in this technique, cell viability can be compromised. Stereolithography is a bioprinting strategy that uses a photo-crosslinking light source to crosslink desired patterns from a bioink reservoir onto a movable platform [7]. This modality is highly tunable and prints in a layer-by-layer manner with relatively high fidelity and speed, depending on the scale of the desired construct. Utilizing these diverse bioprinting strategies, tissue-engineered constructs can be fabricated and tailored at rapid rates with defined, consistent structures and properties, hence providing multidisciplinary platforms for research and biological exploration.

Bioink printability, i.e., the ability to deposit/form stable and viable volumes of material, plays a critical role in the fabrication process. Characterization, tuning, and optimization of bioprintability require an in-depth understanding the flow behavior of the bioink, such as viscosity and shear thinning [8]. To date, a variety of bioink solutions have been commercialized in a variety of biomedical applications (Table 1).

It is a general requirement that bioinks should be flowable and deformable in order to be bioprinted along with being biocompatible and biodegradable [16]. After printing, the bioink should be stable and retain the desired shape and architecture according to the design model. Whether the crosslinking mechanisms are physical and/or chemical, the structural conservation must be compatible with living functional cells and able to sustain the duration of cell culture or in vivo biological environments [16]. The effect of bioink viscosity in 3D bioprinting has been widely studied and has been shown to be one of the most important parameters to consider in devising bioprinting strategies [17]. The review will evaluate the basic printability requirements for the successful selection of bioinks for 3D bioprinting processes.

## 2. Bioink Requirements

Bioprinting must encompass both the qualities of typical 3D printing—reproducibility, structural integrity, and fidelity—as well as using inks that are compatible with living organisms—non-toxicity, degradability, adhesion to cells, and porosity [18,19,20,21,22,23]. Inks with living cells encapsulated, termed bioink, are in conflict as the characteristics that make a stable print, like density or viscosity, are often in direct contrast to sustaining life, as cells need a porous, compliable environment to grow and migrate [24]. Rheological requirements of the bioink vary based on the modality of the bioprinting, i.e., droplet-based or inkjet, laser-based, or extrusion-based printing, with the latter being the most common bioprinting method [20,24,25,26]. Inkjet bioprinting lays a continuous stream of small sized droplets to form the 3D structure. However, this process is usually time consuming and not efficient for large (clinical)-scale tissue manufacturing [22]. Laser-based bioprinting uses a precise laser beam to solidify the construct within a basin of bioink, but may damage the cells with heat [26] and is relatively slow [25]. Extrusion-based bioprinting has a morphology of a filament or string of low viscosity during the print which solidifies once on the print surface holds its shape and supports layering [22]. One main impediment in this method is that the applied extrusion pressure exposes the cells to a noticeable level of (shear) stress as they pass through the syringe and nozzle (needle) which can cause damages [23,25,27]. To limit this stress, the bioink must show less viscous behavior [28], but the risk of this is deformation, collapse, and pore closure which will in turn lower the fidelity and resolution [24].

Hydrogels are a common extrusion-based bioink, because they can encapsulate and support different types of cells and signaling molecules [18,25]. Natural hydrogels are more bioactive, but their synthetic counterparts tend to be more cost efficient and yield more consistent material properties [25]. Most bioinks, including hydrogels, are stabilized with crosslinking strategies to preserve the shape and mechanical integrity of the 3D-printed construct. Physical crosslinking utilizes temperature and/or molecular interactions to strengthen the bonds. Chemical crosslinking is typically mechanically stronger than physical crosslinking because it creates covalent bonds between polymer chains but poses the risk of becoming cytotoxic depending on the agent used. Enzymatic crosslinking also forms covalent bonds between polymer chains but is less controlled in the degree of crosslinking [19]. Controlling the degree of crosslinking is important in bio-implementations because it can modify the stiffness of the construct, which can be used to match the affected tissue [29].

### 2.1. Tissue-Specific Bioinks and Their Requirements

**Hard tissue bioinks:** hard (or calcified) tissues are the types that show mineralized, firm extracellular matrices (ECMs) in the body, including different types of bone, tooth enamel, dentin, and cementum [30]. Bone tissues have the strongest mechanical properties in the human body, with the typical stiffness of cortical bone being about 100 MPa and that of cancellous bone being about 3–4 MPa [31]. Materials with high concentration and high viscosity, including ceramics, poly(caprolactone) (PCL), polylactic acid (PLA), beta tricalcium phosphate (*β*-TCP), have been used to generate bioinks for bone tissue bioprinting [32]. However, as most thermoplastic materials require a high processing temperature and do not support live cell printing, hydrogel-based bioinks have become a more favorable choice of current research [33,34,35,36]. Hydrogel-based bone bioinks typically consist of a printable polymeric backbone from gelatin methacrylate (gelMA), hyaluronic acid, or alginate units that is supplemented with several bone-derived and/or osteogenic minerals and factors, such as beta-tricalcium phosphate (*β*-TCP), transforming growth factor-*β*(TGF-*β*), and bone morphogenetic proteins (BMPs) [34,37].

In addition to mechanical strength, bone tissue mimics should replicate the native tissue microstructure and osteoconductivity to support the vascularization of the printed implants. Factors such as osteoinduction and osteopromotion have a great impact on developing bioprinted functional bone scaffolds. The recruitment of immature bone cells in bioprinted constructs and their stimulation can lead to the formation of preosteoblasts, which can in turn induce osteogenesis, a vital part in the bone healing process [38]. Therefore, biological molecules such as BMP and growth factors (VEGF, TGF, RGDs) are often added into the bioinks to induce bone signaling. Mineral particles including silica, borate, calcium and phosphate are also blended with ECM components and multiple types of cells, such as osteoblasts, mesenchymal stem cells (MSCs), and endothelial cells (ECs), in the bioink to promote osteoinduction and to replicate the dynamic bone microenvironment [33,39,40]. In addition, to facilitate the characterization and functional evaluation of the printed bone grafts, radioactive pharmaceuticals ^99m^*T*c and ^18^F have been reportedly used to label BMP-2 in bioinks to enhance the detectability of bone repair and regeneration in single photon emission CT (SPECT) and positron emission tomography (PET) imaging [41,42,43].

**Soft tissue bioinks:** the bioprinting of soft tissues such as neural, lung and cardiac tissues has distinct and specific requirements for the bioinks. Hydrogel-based bioinks which show relatively low shear stress levels under modest pressure are commonly used for their ideal printing fidelity and capability to maintain in vitro and in vivo cell viability [44,45,46,47,48]. Vascularization is a critical consideration for the bioprinting of many tissues, particularly for cardiac tissues, due to the notably high blood vessel density [49,50,51]. Sacrificial inks such as pluronic and gelatin are often incorporated in the printing process to create hollow channels at diameters ranging from micrometers (capillaries) to centimeters (arteries). Optimal cardiac-specific bioinks should exhibit an elastic modulus ranging from 1 to 16 kPa according to the contractile function of the cardiomyocytes (CMs), and should support the matrix remodeling by the printed cells to achieve intracellular connectivity and high packing density [52,53,54,55,56,57,58]. Therefore, fast-acting crosslinking reagents with negligible cytotoxicity have been included in the bioinks to facilitate gentle crosslinking processes and to provide tunable mechanical properties and degradation profiles of the printed constructs [59].

To achieve the specific functionalization of bioinks, biological and chemical modifications were also employed to generate optimal printable formulations. Small molecules and ECM factors such as BMP2/BMP4 and Wnt inhibitors (IWP2) are often used in cardiac tissue bioinks to promote CM regeneration [60,61,62,63,64,65,66]. ECM proteins such as collagen, connexins, cadherins can be incorporated to promote cell attachment, proliferation and matrix remodeling [57,67,68,69,70,71]. Secreted small molecules such as transforming growth factor beta (TGFβ), interleukin (IL)-1, IL-6, tumor necrosis factor alpha (TNFα), endothelin 1 and angiotensin II can also promote the maturation and vascularization of the printed cardiac tissues [72,73]. Different cell types including MSCs, ECs and smooth muscle cells (SMCs) can be co-encapsulated in the bioinks, along with CMs, to provide 3D scaffolding systems that closely recapitulate the complex heart tissue. A main challenge in bioprinting such highly cellularized bioinks which is a current research focus is the negative impact of large cell numbers on the rheological properties and printing fidelity [74,75,76,77]. In addition, further research is needed to design and develop non-invasive imaging modalities that enable the monitoring and evaluation of printed tissues, possibly by incorporating various contrast agents in the tissue-specific bioinks to enhance the imaging signals of magnetic resonance (MR) or computed tomography (CT) imaging [78,79].

Bioinks for other, non-cardiac, soft tissues also require specific properties to achieve both physiological relevance and bioprintability. For instance, hepatic lobules have a complex organization of hepatocytes, hepatic stellate cells, and ECM. Thus, liver bioinks are required to contain functional ECM components, such as collagen type I, to allow for native cell interaction, reorganization, and liver function maintenance [80,81]. Neural tissue bioprinting employs bioinks with ideal conductivity, biological cues, and growth factors, including VEGF, for better neural cell growth and differentiation. Lung tissue bioprinting might combine multiple bioinks in order to create the air–blood barrier architecture, while skin bioinks need to support the co-culture of various cell types to recapitulate the multi-layered tissue structure [82,83].

### 2.2. Specific Bioink Requirements for Clinical Applications

The patient specificity of bioengineered implants has been increasingly designed and examined in various tissue engineering and regenerative medicine applications. The coupling of the 3D bioprinting technologies with advanced medical imaging modalities, such as MR and CT, have enabled major advancements in the fields of precision and personalized medicine [84]. For extensive use in clinical applications, the bioink material, like other implant biomaterials, must demonstrate high levels of biocompatibility, inertness, mechanical stability, and the ability to be remodeled by cells. There are already many identified clinical applications for 3D printing and bioprinting, especially in orthopedics, orthodontics, and other hard tissue applications. Biomaterials such as PLA and PCL are mainly utilized as bioinks in the clinical printing applications, due to their excellent mechanical properties, biocompatibility, and degradation rate [85]. There is, however, a demand for the development of highly tunable and versatile novel bioinks that can be used in soft tissue applications. Bioinks that contain cellular and antigenic components at times often elicit an immune response [86]. Tissue decellularization has been used as an effective method to prepare highly bioactive, patient-specific bioinks for various tissue bioprinting applications [87]. Naturally derived polymers such as alginate, fibrin, HA, collagen, and gelatin are highly printable using extrusion-based bioprinting. They offer a low donor-site morbidity and invoke a low immune response [88]. However, in vivo, they often undergo rapid degradation and/or remodeling which decreases their mechanical stability and fidelity over time.

## 3. Printability and Bioprintability

In 3D printing, printability generally refers to the ability of a material to be fabricated in a layer-by-layer sequence into a 3D object with well controlled design [89]. For biomedical applications, further criteria are needed to consider a material for 3D fabrication since these systems need to be able to host and maintain cell proliferation. For instance, even though thermoplastic materials like thermoplastic polyurethane (TPU) and PCL are biocompatible, they have only been used as support structures which can be sacrificed post printing, since they have a high melting temperature (>90 °C) which would be detrimental to cell cultures [90,91,92]. In addition, many 3D printable synthetic polymer materials cannot be used in 3D bioprinting due to their low cell affinity [93] and high toxicity [94] which can trigger inflammatory or other adverse response in clinical application [95].

As alternatives to traditional plastics and metal inks, bioinks have been developed for specific 3D bioprinting applications with clinical targets including different artificial organs and tissues. Due to their low fabrication temperature (~37 °C or lower), only a limited subset of polymer materials can be used as bioinks for 3D printing processes since they need to satisfy several important criteria for printability. These include: (i) rheological properties, i.e., the ability of the material to deform, flow, and be precisely controlled to form a 3D shape [96]; (ii) viscosity [97]; and (iii) surface tension [98]. These properties are strongly dependent on the printing techniques. For instance, the printability of bioinks composed of glycerol–water solutions is determined by a combination of viscosity, surface tension, and the density of the ink fluids in the fabrication method like inkjet printing [99]. In inkjet bioprinting, it is important that the material needs to have a rheopectic behavior, thus allowing the gel-like material to form a droplet shape after ejection due to the increasing viscosity [19]. Thus, only bioinks with low viscosities in the range of 10 mPa·s are desirable in this bioprinting modality [100,101].

**Figure 1:**
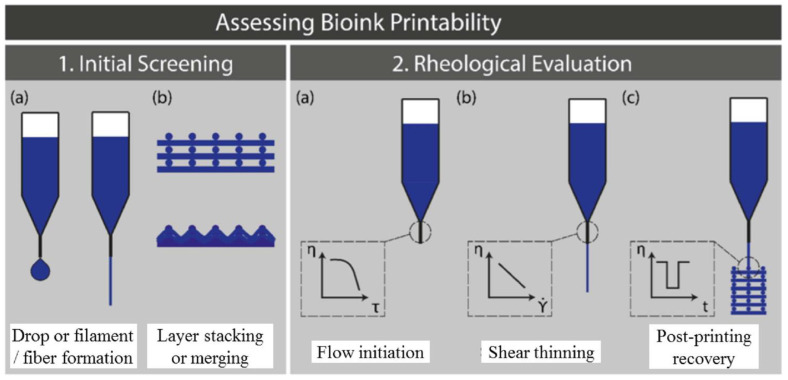
Schematic illustration of the workflow to assess the bioink printability for extrusion-based bioprinting [96].

Other bioprinting methods such as extrusion-based printing requires the bioink to exhibit thixotropy properties, i.e., reducing viscosity under applied shear stress. This requirement is mainly due to the fact that microextruder bioprinters eject larger filament-like structures than the droplet sizes (Figure 1) [26]. As a result, commonly used non-Newtonian fluids like hydrogels are the preferred bioink type for extrusion-based bioprinting [19]. Another advantage of extrusion-based bioprinting is that it can print highly viscous cell-laden bioinks by adjusting the air pressure during the pneumatic deposition [98,102,103]. One disadvantage of this method is that the shear thinning property of bioink can affect the cell viability during printing [104].

Another common type of bioprinting is laser-assisted printing which can be divided into two categories: laser-induced forward transfer (LIFT), and photo-induced polymerization known as stereolithography [19]. In LIFT bioprinting, a pulsed laser is used as an energy source to induce a jet formation to the substrate. This technique requires the bioink to contain a viscosity in the range of 1 to 300 mPa·s [5,98]. Due to these relatively low viscosity values, it is important that the 3D structure is adequately crosslinked post printing to improve the mechanical integrity [5]. For stereolithographic applications, photosensitive bioinks are used [19]. An advantage of this printing technique is that it can utilize high-viscosity bioinks, while minimizing the mechanical stresses on the encapsulated cells that is typically generated by more viscous hydrogels, thus enabling higher cell viability and functionality [105]. 

## 4. Bioprintability Assessment 

The assessment of bioprintability requires comprehensive measurements to be performed on the created constructs at multiple scales. Printing fidelity is a critical parameter that has been frequently used to describe the bioprintability of bioinks. Fidelity defines the level of structural differences between the design and actual prints [26]. In general, higher printing fidelity indicates a lower level of structural variability, demonstrating a closer match between the designed and printed structures. Lower fidelity values illustrate larger differences between the design and bioprints. Due to the liquidity and inherently low mechanical strength of hydrogel-based bioinks, it has always been challenging to build 3D hydrogel constructs with high printing fidelity [98].

**Figure 2:**
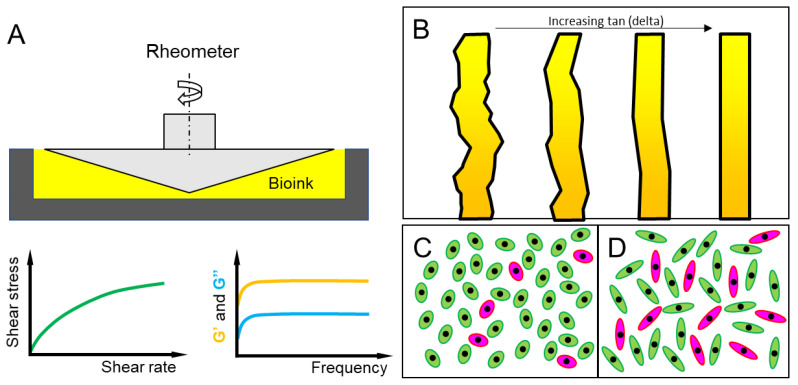
Rheological properties and their effects on bioprinting fidelity and cell viability. (**A**): Cone-and-plate rheometer and its application for evaluating the flow behavior and viscoelasticity of a bioink. (**B**): Influence of loss tangent on printed strand fidelity. (**C**–**D**): Influence of bioink viscosity on cell viability after bioprinting. A higher cell viability is found within lower viscosity (**C**) versus higher viscosity (**D**) bioinks. Green represents live cells and red represents dead cells.

Since fidelity ultimately determines the precision of the spatial control of bioink deposition, several strategies have been developed aiming to preserve or enhance the fidelity of bioinks in bioprinting processes. These strategies either rely on bioink modifications, or in situ crosslinking, or sometimes both. Bioink modification focuses on the rheological properties of the material, with the most efforts centered around viscosity [102]. A bioink with greater viscosity is likely to provide stronger mechanical support and resistance against structural deformation (e.g., due to gravity) during bioprinting, resulting in improved printing fidelity [24]. However, the excessive viscosity of bioinks often restricts the survival and functionality of cells, reducing their bioactivity when cells are encapsulated [106]. Additionally, bioinks with higher viscosity require a greater printing pressure to maintain the given printing flow rate, which in turn could cause increased cellular damages [107,108]. Bioinks with lower viscosity are more suitable for cells but they often face the issue of suboptimal printing fidelity and resolution. Therefore, a delicate compromise between the printing fidelity and cytocompatibility for a bioink should be established to achieve optimal tissue bioprinting efficiency (Figure 2). Higher viscosity, however, may not necessarily guarantee the high fidelity printing of soft tissue constructs [109]. Other bioink properties, such as storage (G’) and loss moduli (G’’), also play significant roles in bioprintability [110]. The storage modulus indicates the elastic behavior of the bioink while the loss modulus represents the liquid aspect. Typically, a bioink with a larger G’ value compared to G’’ provides a higher printing fidelity due to its increased mechanical stiffness. The bioprinting fidelity is identified according to the ratio between G’ and G’’ (also known as the loss tangent, *δ*) which is highly dependent on the type of the bioink polymer [111,112].

Rheological behavior of a bioink can be regulated using both physical and chemical methods. The most straightforward way is to adjust the concentration of hydrogel materials or to blend them with other materials [113]. However, regulating the rheological behavior is usually time consuming and may not meet the biocompatibility requirement. Therefore, the second group of strategies, the in situ crosslinking methods, were used. In situ crosslinking is known as “bioprinting while crosslinking”, which is used to stabilize the extruded bioinks during the printing process [114]. It significantly improves the mechanical stability of the extruded bioinks in shape, thus maintaining fidelity. Depending on the properties of bioinks, various crosslinking approaches have been tested. The crosslinking of thermosensitive hydrogels is triggered by the control of printing temperature [111]. The printing fidelity of these hydrogel bioinks relies on their mechanical properties and normally requires additional crosslinking due to their reversibility. For bioinks that can be crosslinked through ionic bonding, a crosslinking medium (bath) can be used to promote the material stabilization, which significantly reduces the spread of the bioink, thus preserving a higher printing fidelity [115]. This method normally requires a stringent adjustment of the crosslinking bath to avoid printing failures, which are introduced by the buoyance of the crosslinking agent [116].

In situ crosslinking approaches are also applicable for photo-crosslinkable bioinks [117]. During this procedure, a light source is incorporated in the printing system for initiating the polymerization. By controlling the light intensity or exposure time, the printing fidelity of bioinks can be tuned. Due to limited crosslinking rates, maintaining the fidelity of printed filaments is not ideal via in situ photo-polymerization, especially when bioinks with low viscosity are extruded [118]. The other drawback of this method is that it may introduce extra exposure to the first few deposited layers, leading to excessive stiffness and increased overall cell damage. An alternative method partially stabilizes the dynamic flow of bioinks within the light-permeable needle before extruding out. It creates filaments with improved stability and uniformity and allows the printing of low-viscosity hydrogel inks via the adjustment of light intensity and exposure time [119]. This unique approach requires a light permeable needle which is normally custom-made from glass or silicon. An elaborate adjustment of the light source is also necessary to ensure the uniform flow of bioink.

### 4.1. Experimental Methods for Bioprinting Fidelity Assessment

**Figure 3:**
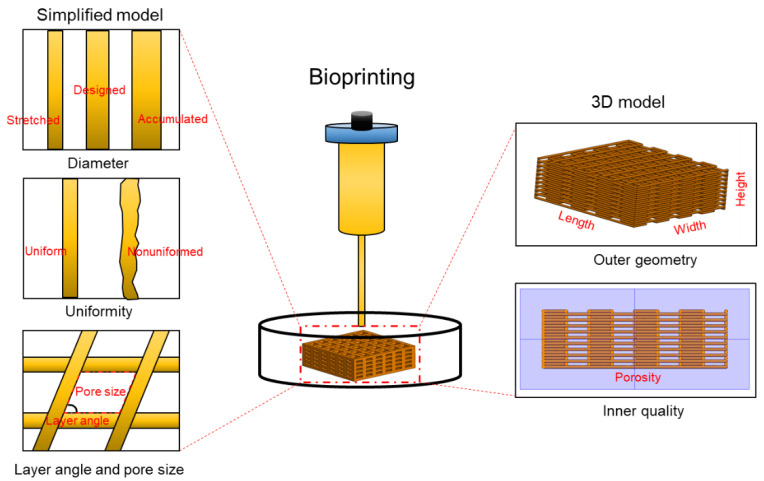
Geometrical parameters involved in the 3D bioprinting fidelity assessment.

Printed filaments (strands) act as the building blocks for 3D structures in extrusion-based bioprinting, and their shape fidelity (stability) directly affects the printing fidelity of the structure in its entirety. Fidelity has been prevalently assessed through visual inspection after bioprinting-based on the microscopic evaluation of printed single filaments or layers as models for the assessment [27]. These evaluations consist of both qualitative and quantitative analyses of the geometrical features of printed models (Figure 3). The geometric characteristics that have been commonly used for fidelity evaluations include filament diameter and uniformity [112,120]. The diameter describes the actual size of filaments, and its similarity to the designed model can report on the bioprinting fidelity. Uniformity quantifies the length of the printed filaments and is used to determine the consistency of the bioprinted filaments in comparison to the designed model. Filaments with straight edges show higher uniformity, and hence, printing fidelity. On the contrary, printed strands with bumpy or ragged edges would yield lower bioprinting fidelity. The ability of bioprinted filaments to avoid collapse and maintaining their shape has also been identified as a particularly important index for bioprinting fidelity evaluation, when dealing with much softer materials (hydrogels) [24]. In comparison to the size and uniformity assessments, which are typically obtained from single filaments or layers in the *x*–*y* horizontal plane, the ability of filament to resist gravity and falling in an overhanging structure is assessed along the *x*–*z* or *y*–*z* vertical planes. The bioprinting fidelity, which is quantified by the mid-span deflection angle, relies on the yield stress of filament under gravity, as well as the structural gap size. This highlights the role of the yield stress of a bioink to maintain the printed filaments from deformation as well as the structural design to reduce the collapse along the vertical direction.

The assessment of printing fidelity normally characterizes the outer geometry (e.g., height, width, and length) and the inner structure (e.g., layer angle, pore size, porosity, and printing angle) of 3D constructs. Like filament evaluation, the outer geometry is measured based on visual/microscopic inspection, and the printing fidelity is evaluated according to the comparison of size and shape between the bioprinted structures and their CAD designs [111,112]. Layer angle and pore size, which are created by the layer stacking, are often measured based on simplified models [22,121]. These models act as the unit element to form the entire structure following repetitive stacking, thus recapitulating the basic characteristics of actual 3D structures. However, they are not able to quantify the deformation of bioprints along the *z* direction and are therefore unable to represent the inner quality of integrated bioprints after layer assembly. For this purpose, imaging techniques such as micro computed tomography (µCT) have been used to rebuild the entire 3D structures of bioprints and analyze the porosity [122,123]. One major issue for this technique is that the post processing of bioprinted structures is normally required due to the relatively low density and weak X-ray attenuation of hydrogel materials [123]. Such treatment on hydrogel-based constructs may partially damage the printed structure and result in compromised accuracy. Alternatively, magnetic resonance imaging (MRI) and ultrasound imaging (UI) have shown great potential for the characterization of the hydrogel constructs [124,125]. However, both techniques face constraints to achieve high spatial reconstruction within feasible scan times. A promising imaging technique that relies on synchrotron X-ray propagation-based imaging in combination with CT imaging (SR-PBI–CT) has recently been developed for the visualization and analysis of hydrogel-based 3D bioprinted constructs [114]. Through introducing the X-ray refraction signal, the PBI–CT technique shows promise in the visualization of low-density hydrogels due to the much larger refractive index variations. Combined with synchrotron radiation which is highly brilliant and a coherent light source, SR-PBI-CT has presented great capacity in visualizing hydrogels without the need for pre or post processing. 

**Figure 4:**
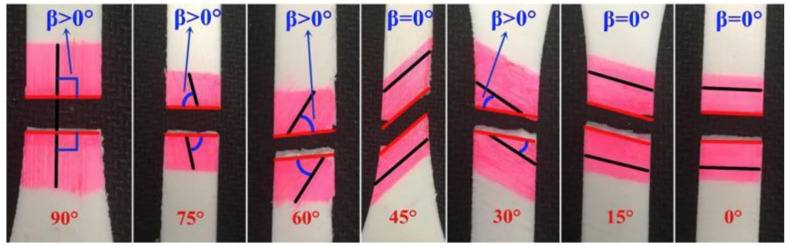
Ultimate tensile strength testing of polylactic acid (PLA) materials and their respective printing angles. Reprinted with permission from [126].

The printing angle is another prominent parameter that has been studied in 3D printing and bioprinting applications, mostly to test its effect on print fidelity and the ultimate tensile strength (UTS). Yao et al. discovered that the UTS of the 3D printed PLA materials decreases with the decrease in the printing angle, from 90° to 0° (Figure 4). They reported a significant difference in the measured UTS (52.29%) between the 0° and 90° printing angles [126]. The theoretical models utilized in this study can aid in extracting important information on overall printing fidelity, to determine whether a critical defect printing angle exists.

### 4.2. Mathematical Models for Printing Fidelity Characterizations

The size of deposited bioink filaments is not only determined by the properties of bioink and the crosslinking procedure, but also heavily depends on the printing process control [127]. To create filaments with uniform size and faithful to the designed model, most printing attempts follow a trial-and-error approach, through a series of re-adjustments of the printing process parameters, such as pressure, temperature and moving speed, and evaluating the effect on fidelity [128]. Such approaches are typically laborious and time consuming, requiring many repetitions which could also waste significant quantities of bioinks (and biological contents). To avoid these “hit or miss” strategies, a simple mathematical model has been developed based on the relationship between the flow rate and printing speed [129]. It is based on the prediction of the bioprinting flow rate, assuming that the bioink is incompressible [108]. If the effects of swelling and gravity can be ignored, the diameter of printed filament can be expressed as

(1) $$D_{p}=\;\sqrt{\frac{4Q}{\pi V_{p}}}$$

where $D_{p}$ represents the diameter of the printed filament, Q is the volumetric flow rate of bioink, and $V_{p}$ represents the printing speed in horizontal plate. The large flow rate associated with lower printing speed increases the filament diameter, while a small flow rate combined with higher printing speed reduces the size. Using this equation, the geometry of printed filament can be readily regulated following the steps: 1) based on the printing resolution, selecting the printing needle with the appropriate size; 2) based on the needle size, establishing printing models with designed filament size; 3) adjusting the flow rate and printing speed to achieve a calculated filament size (using equation (1)) that matches the predetermined size; and 4) bioprinting the filament and measuring its actual size. Incorporating this mathematical model into pre-printing planning could therefore help remarkably to adjust the starting printing parameters more efficiently and accurately, and to achieve the targeted bioprinting fidelity. 

## 5. Bioink Support for Cells and Other Biological Factors

As 3D bioprinted constructs are often fabricated with cells, it is crucial for the bioinks to accommodate proper cell viability and function, while maintaining adequate mechanical properties for bioprintability [26]. However, many studies that investigated the effects of bioink properties suggest that there is a trade-off between the bioprintability and biocompatibility of the bioinks [27]. Often, adjusting parameters that can reinforce printability, such as increasing concentration or lowering the printing temperature, induces a harsh environment for cells to survive. Consequently, this compromise narrows the biofabrication window, imposing difficulties in the simultaneous achievement of adequate print fidelity and cell viability.

To address the challenge of cell survival during printing, several modifications have been made to bioinks to enhance their bioactivity during and after printing. Many inert hydrogels have been employed as bioinks due to their biocompatibility and mechanical similarity to the natural ECM [21]. However, the cells tend to show limited cell attachment, proliferation, and functionality in such materials [19]. This is contributed to the fact that many hydrogel bioinks, despite their non-toxic nature, lack the essential cell-supporting components such as cell binding sites. To address this issue, cell binding peptides can be incorporated into the bioinks. Studies have shown that bioinks with RGD peptides result in improved cell viability and proliferation [130,131]. Furthermore, hydrogels with intrinsic cell binding sites are commonly used. Such natural materials, including fibrin and gelatin-based bioinks, have inherent cell binding sites and can allow cell adhesion and proliferation without the need for modifications [98]. 

Bioinks can further enhance the functionality of cells by entrapping growth factors and other signaling proteins into the matrix. This allows the targeted delivery of important functional molecules to the cells, thereby creating a favorable microenvironment for specialized cellular activities [21]. For instance, Skardal et al. demonstrated the use of a hyaluronic acid and gelatin hydrogel composite infused with a liver ECM solution that contained a mixture of liver-specific growth factors [132]. Hepatocytes printed in the liver-specific hydrogel bioink were able to survive over 28 days and showed increased liver functions compared to 2D cultures.

**Figure 5:**
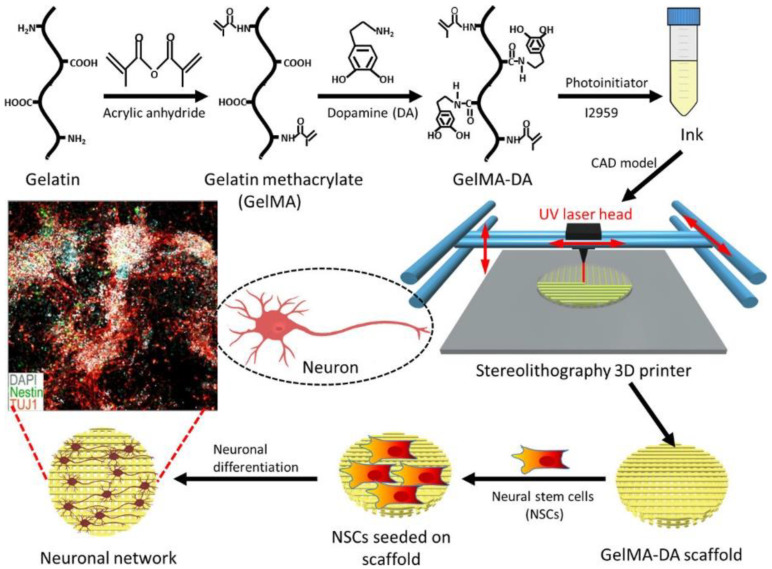
Schematic of a 3D bioprinted matrix utilized to promote neural cell support for tissue regeneration. Reprinted with permission from [134].

The functionalization of bioinks with small molecules or proteins is another way to direct cellular responses via cell–material interactions. The immobilization of proteins in bioinks enables sustained exposure and subsequent responses such as stem cell differentiation [133]. In one study, dopamine was crosslinked to the gelMA bioink to promote neural differentiation (Figure 5) [134]. They found that gelMA functionalized with dopamine provided a favorable environment for neuronal stem cell growth and differentiation into specialized cells compared to the gelMA alone. More recently, novel methods to modify bioinks have been developed to incorporate various protein therapeutics or ECM components to control cellular activities via cell–matrix interactions [135,136]. These strategies are expected to further advance bioink designs to provide support for cell survival and functions.

Decellularized ECM (dECM) has also gained attention as natural, potentially patient-specific bioinks retain most natural ECM constituents and growth factors. Therefore, dECM bioinks not only present the mechanical properties appropriate for both cell support and printing, but can also provide the microenvironment suitable for tissue-specific cellular functions [137]. In a recent study, Lee et al. printed mesenchymal stem cells and hepatocytes using a liver dECM-based bioink [138]. It was demonstrated that the liver dECM has adjustable printability and provides a biochemical environment that can stimulate stem cell differentiation as well as enhanced hepatocyte functions.

## 6. Post-Print Bioink Properties

To obtain functional bioprinted constructs with high fidelity as well as appropriate cell viability and function, the maintenance of fabricated constructs after the printing is just as important as the various considerations during the bioprinting process. The first thing to consider is preserving the shape fidelity throughout the crosslinking processes, which are traditionally categorized into chemical and physical methods. Chemical crosslinking introduces covalent bonds, while physical crosslinking is generated by electrostatic, hydrophobic or hydrogen bonding [139]. The most frequently used chemical crosslinking methods include photo-crosslinking, enzymatic linkage, amidation, and Michael addition reaction. On the one hand, supramolecular networks, thermally induced hydrogels and cyclodextrin-based materials are largely explored examples of physical crosslinking. The time of introducing crosslinking is critical for maintaining intricate printed structures and should be planned along with the rest of the bioprinting process control. For instance, the viscosity of materials used for inkjet bioprinting and orifice-free bioprinting is generally low and often lacks the ability to self-stand after being deposited onto the substrate. In such modalities, therefore, instantaneous crosslinking should be implemented. Extrusion-based bioprinting, on the other hand, is able to handle more viscous bioinks, with greater yield stress, that are able to sustain structural integrity for longer periods before crosslinking [98]. Higher yield stress often requires higher concentration of materials, which might compromise biocompatibility [140]. To overcome the dilemma between shape maintenance and appropriate bioink concentration, strategies such as pre crosslinking, printing into supporting self-healing hydrogel [141], and embedded bioprinting methods are proposed [142]. 

Aside from the timing of the crosslinking step, the degree of induced molecular interactions and entanglement affect the fidelity and outcomes of functional printed constructs by influencing hydrogel pore sizes, final mechanical properties, swelling behavior, as well as degradation rate. Thus, protocols have been proposed for measuring the crosslinking degree of printed constructs. The equilibrium swelling test is a traditional assay to determine the concentration of crosslinkers per unit volume, which could be calculated from the swelling degree by measuring the weight of the hydrogel under equilibrium swelling state and desiccated state [143]. Another candidate is the uniaxial compression test. The degree of elastically effective physical and chemical crosslinking can be calculated based on the equation derived by Flory et al. from the measured compression stress [144,145]. Solid state nuclear magnetic resonance (NMR) spectroscopy measurement can also be used to assess chemically effective crosslinking [143].

Another essential factor that might influence the fidelity and function of a bioprinted construct for in vitro tissue modeling or in vivo regeneration is the volumetric changes caused by swelling behavior and culture condition variations, including the temperature or pH changes, by which stimuli responsible hydrogels are most influenced [146,147]. Although these events are difficult to avoid and generally have a negative influence on the final fidelity of the printed constructs, more intricate print process design and control can help neutralize some of these effects. Shape expansion caused by swelling could be offset by incorporating temperature-sensitive polymers, such as poly(phenylene oxide), with a hydrophilic-to-hydrophobic transition. Encapsulated cells can remodel the bioprinted structure by fusion and self-assembly processes. Sun et al. developed a model that can predict the time evolution of post-printing morphological structures [148].

In terms of temporal properties in the long term, the degradation behavior of bioprinted constructs can have essential effects on their fidelity and function. Biodegradable or non-degradable materials may be considered bioinks, according to the specific applications. Bioprinted constructs typically exhibit distinct degradation rates in the in vivo versus in vitro settings [149,150]. Degradation can be categorized into hydrolytic, oxidative, enzymatic degradation, and photo-degradation. Material composition, pH, enzymes, construct structure, crosslinking, weathering, physical loading, and other factors can influence the degradation rate [151]. Thus, a desired degradation rate can be adjusted by taking these parameters into consideration.

## 7. Conclusions

Three-dimensional bioprinting is a rapidly evolving additive manufacturing strategy to develop tissue constructs at the clinical and industrial scales, while providing highly tunable and reproducible functionalities. The pioneering works in this field have proven the feasibility of bioprinting a variety of biomaterials and cellular components to form rather complex tissue constructs. Although there have been expansive efforts to advance this field, the lack of standardized methods to characterize the optimal mechanical, rheological, and biological properties of bioinks have hindered particularly the in vivo and clinical applications. As the commercial availability of novel bioink formulations arises, there may be new metrics in place to aid the assessment of bioprinting fidelity. This review provided current state-of-the-art methods, together with experimental and mathematical procedures that have been used to analyze bioprintability and develop optimized bioink solutions for tissue-specific applications. While the experimental methods to assess and tune bioprinting fidelity typically rely on repetitive trial-and-error steps, the emerging mathematical and computational models have shown great promise in improving the efficiency and accuracy of these processes. These theoretical models, however, would still require further enhancement, for instance, to incorporate and carefully assess the cellular and biological factors involved in various bioprinting procedures.

## Figures and Tables

**Table 1:** Commercially available bioinks.

Product	Company	Materials	Advantages
Viscoll	3D Bioprinting Solutions, Moscow, Russia	Type I collagen	Can be supplemented with growth factors, biocompatility, cell printing, rapid polymerization [9]
CELLINK A CELLINK A-RGD	CELLINK, Gothenburg, Sweden	Alginate L-arginine-Glycine-L-aspartic Acid peptide	Cartligae, bone and mesenchymal stem cells, can be used for drug delivery and cell differentiation [10]
GelMA Bio Conductink GelMA A	CELLINK, Gothenburg, Sweden	Gelatin methacrylate GelMA and carbon nanotubes Gelatin methacrylate and alginate	Designed for neural, cardiac, and skeletal muscle cells, facilitates electrical potential, photo-crosslinkable [11]
Fibronectin-functionalized synthetic peptide hydrogel bioink	Regemat 3D, Granada, Spain	Fibronectin	Form nanofibrous network, mimics extracellular matrix, tunable mechanical and chemical properties [12]
3D-Bioplotter HT PCL	EnvisionTEC, Gladeck, Germany	Polycaprolactone	Versatile thermoplastic, bone and cartilage regeneration, biodegradable, excellent mechanical stability, allows for controlled drug release [13]
PhotoHA^®^	Advanced BioMatrix, Carlsbad, CA	Methacrylated hyaluronic acid	Has been used in cartilage tissue applications [14]
Collagen Lifeink^®^ 200		Type I collagen	Superior cytocompatibility, supports cellular remodeling, highly biomimetic [15]
